# Projecting spatiotemporal changes in Soil Organic Carbon stocks in Mexico under coupled socioeconomic and climate scenarios

**DOI:** 10.1371/journal.pone.0343012

**Published:** 2026-02-20

**Authors:** Yuan Wang, Xiaoxiong Li, Sha Liu

**Affiliations:** 1 Faculty of Geography and Resource Sciences, Sichuan Normal University, Chengdu, Sichuan, China; 2 Nanjing Gago Group, Nanjing, Jiangsu, China; 3 Yunnan Provincial Key Laboratory of Soil Carbon Sequestration and Pollution Control, Faculty of Environmental Science and Engineering, Kunming University of Science and Technology, Kunming, Yunnan, China; Nepal Agricultural Research Council, NEPAL

## Abstract

Soil organic carbon (SOC) sequestration is a pivotal strategy for achieving global climate goals, yet current projections often rely on static landscape assumptions that fail to capture the complex interplay between climatic forcing and socioeconomic development. This limitation is particularly acute in Mexico, where rapid agricultural transformation threatens to destabilize historic carbon sinks. To bridge this gap, we projected spatiotemporal SOC dynamics (2021–2100) across Mexico’s diverse ecoregions by coupling a calibrated Random Forest model (R^2^ = 0.45) with downscaled climate and dynamic land-use data under four Shared Socioeconomic Pathways (SSPs). We identified a current baseline mean SOC stock of 2.96 kg C/m² and revealed stark, pathway-dependent future trajectories. While sustainable and intermediate pathways (SSP126, SSP245, SSP370) stabilize or slightly increase national stocks (~2% gain), the fossil-fueled development scenario (SSP585) drives a continuous decline below baseline levels. Novel analysis of agricultural systems reveals a critical tipping point: under SSP585, the proportion of degrading cropland escalates from 36.7% to 63.2%, transitioning the sector from a net carbon sink to a net source after 2070. These findings provide quantitative evidence that future SOC stability is not merely a climatic outcome but is critically determined by policy-driven land-use choices, offering a data-driven basis for immediate intervention in threatened agricultural zones.

## Introduction

The terrestrial carbon pool, particularly soil organic carbon (SOC), represents a fundamental nexus in the Earth’s biogeochemical cycles, orchestrating crucial ecosystem functions including primary productivity, pedological stability, biogeochemical transformations, and ecosystem resilience [1]. The paradigm shift in climate science has elevated SOC’s significance beyond traditional ecological services to a pivotal role in anthropogenic climate change mitigation strategies and the achievement of United Nations Sustainable Development Goals (SDGs) [2]. Contemporary estimates indicate that global soil carbon pools, predominantly within the upper 100 cm, sequester approximately 1,500 Pg of carbon, constituting a repository that quantitatively supersedes atmospheric carbon pools by a factor of two and terrestrial biosphere stocks by a factor of three. This substantial carbon repository exhibits remarkable sensitivity, where perturbations in SOC stocks can trigger cascading effects on atmospheric CO_2_ concentrations [3]. The recognition of SOC’s multifaceted significance has catalyzed intensive scientific inquiry across multiple spatial and temporal scales. Nevertheless, methodological heterogeneity and data inconsistencies persist as significant impediments, particularly in the realm of long-term spatiotemporal SOC trajectory modeling under various climate change scenarios [4]. These knowledge gaps underscore the imperative for rigorous investigation to elucidate SOC dynamics and their implications for global carbon cycle perturbations at varying geographical contexts.

The quantification and monitoring of SOC stock and their spatial distributions constitute foundational elements in terrestrial carbon cycle research. The resource-intensive and temporally demanding nature of direct SOC measurements has necessitated alternative methodological frameworks among United Nations Framework Convention on Climate Change signatories for carbon stock assessment and flux monitoring [5,6]. The methodological spectrum for SOC quantification encompasses approaches ranging from IPCC-standardized default parameters to sophisticated regionally-calibrated process-based or empiric models [7]. Contemporary national-scale SOC modeling frameworks integrate multifaceted data streams, synthesizing landscape-level carbon dynamics, geospatial information on topography and parent material, and systematic soil inventory databases. Such integrative approaches aim to capture the inherent spatial heterogeneity and temporal dynamics of SOC across diverse ecological matrices and anthropogenic land-use gradients [8]. Enhanced mechanistic understanding of SOC behavior and refined predictive capabilities emerge as imperative prerequisites for evidence-based policy formulation in terrestrial carbon management and climate change mitigation initiatives [9]. The continuous refinement of SOC estimation methodologies and modeling frameworks facilitates more precise carbon accounting protocols, underpins sustainable agricultural practices, and augments ecosystem resilience against unprecedented environmental perturbations.

The diverse, rapidly changing climatic and physiographic landscapes of Mexico present a compelling case study for investigating SOC dynamics under anthropogenic forcing. The nation is characterized by significant climatic gradients, from arid and semi-arid regions to humid tropics, and is projected to experience accelerated climate change impacts throughout the 21st century [10]. However, these ecosystems are under dual pressure: alongside climatic aridification, extensive agricultural landscapes are undergoing rapid transformation, threatening to destabilize historic carbon sinks.

Although extensive investigations have elucidated historical SOC dynamics across Mexico’s diverse edaphic gradients using various inventory protocols [8,10], critical knowledge gaps persist regarding future trajectories. A major limitation in contemporary SOC modeling is the methodological inconsistency and the reliance on “static landscape” assumptions [4]. Many existing frameworks project SOC responses to climate perturbations but fail to explicitly integrate dynamic, policy-driven Land Use/Land Cover (LULC) changes. This disconnect is particularly problematic for developing economies, where socioeconomic choices often override climatic signals in determining carbon storage.

Bridging this “SOC-climate-land use” modeling gap is imperative for robust carbon management [22]. Future research trajectories necessitate the development of sophisticated modeling architectures capable of capturing the intricate spatiotemporal dynamics between SOC pools and climatological variables, while explicitly incorporating key geographical determinants such as topography, parent material, and dynamic anthropogenic management regimes [11]. Specifically, integrating the Shared Socioeconomic Pathways (SSPs) is essential to resolve how different development scenarios will spatially alter soil resilience, a dimension often overlooked in climate-only projections [16].

Enhanced predictive capabilities and mechanistic understanding of these coupled dynamics will facilitate evidence-based policy formulation [9]. By quantifying the divergence between different SSPs, this research provides the necessary data-driven basis for targeted interventions, advancing sustainable terrestrial ecosystem management and actualizing national carbon neutrality aspirations and Sustainable Development Goals [2].

## Methods

### 2.1 Study area

**Fig 1:**
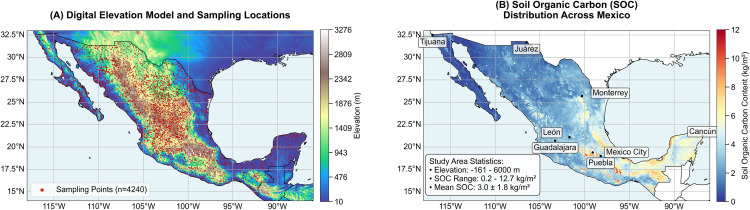
Spatial distribution of soil sampling points (A) and Baseline map (B) of SOC stock under current climate conditions across Mexico.

The study was conducted across the national territory of Mexico, a region characterized by profound climatic and topographic heterogeneity (Fig 1A). The landscape encompasses diverse ecoregions, ranging from arid and semi-arid deserts in the north to humid tropical forests in the southeast, shaped by complex orography including the Sierra Madre Occidental and Oriental ranges. This environmental heterogeneity generates strong gradients in temperature, precipitation, and vegetation, which are primary drivers of Soil Organic Carbon (SOC) distribution. Special emphasis was placed on agricultural landscapes, which comprise approximately 13.6% (26.7 million hectares) of the national territory and are distributed across these varied environmental zones [12].

### 2.2 Data acquisition and processing

#### 2.2.1 Soil organic carbon inventory.

The pedological dataset was retrieved from the INEGI soil inventory archives [8]. To ensure temporal consistency and mitigate confounding effects from historical land-use shifts, we restricted the dataset to 4,240 georeferenced observations collected during the 1991–2010 systematic sampling campaign (Fig 1A). SOC quantification adhered to USDA Soil Taxonomy protocols, predominantly using the Walkley-Black oxidation method. The primary response variable, Soil Organic Carbon Stock (SOCS, kg C/m²), was standardized to the 0–30 cm topsoil depth using measured concentrations and bulk density data.

#### 2.2.2 Environmental covariates.

We compiled a suite of environmental predictors at a 1-km spatial resolution, stratified into climatic, topographic, and edaphic categories to model SOC distribution. Climatic inputs consisted of nineteen bioclimatic variables (BIO1–BIO19) from the WorldClim baseline (1970–2000), prioritizing metrics that capture physiological growth windows such as Mean Annual Temperature and Annual Precipitation. Topographic parameters, including elevation, slope, aspect, Topographic Wetness Index (TWI), and solar radiation, were derived from the SRTM 90m Digital Elevation Model. These were complemented by edaphic covariates—specifically baseline soil texture (sand, silt, clay fractions) and pH—sourced from the ISRIC SoilGrids database to account for parent material influence.

#### 2.2.3 Future climate and land use scenarios.

For future projections (2021–2100), we utilized downscaled global climate and Land Use/Land Cover (LULC) data from Zhang et al. [16]. These datasets provide spatially explicit projections under four Shared Socioeconomic Pathways (SSPs): SSP126 (Sustainability), SSP245 (Middle-of-the-road), SSP370 (Regional Rivalry), and SSP585 (Fossil-fueled Development). The LULC projections were generated using the cellular automata-based PLUS model, dynamically downscaled from Global Change Assessment Model (GCAM) demands.

## 2.3 Modeling framework

### 2.3.1 Model configuration.

**Fig 2:**
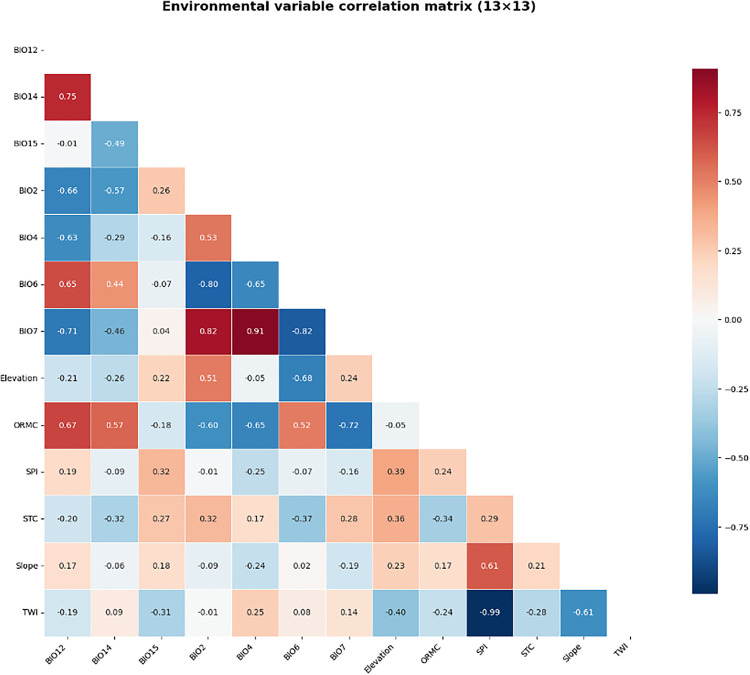
Pearson correlation matrix of environmental predictor variables.

We developed a Random Forest (RF) regression model within the Google Earth Engine (GEE) platform [13]. Feature analysis indicated high collinearity among specific bioclimatic variables (Fig 2). However, the predictor suite was retained. The theoretical justification is twofold: first, the ensemble decision-tree architecture of RF is inherently robust to multicollinearity [21]. Second, statistically correlated variables often delineate distinct ecological mechanisms essential for resolving non-linear SOC dynamics. For instance, while Mean Annual Temperature (BIO1) represents general thermal energy, Temperature Seasonality (BIO4) captures thermal variability that shapes microbial resilience, and Minimum Temperature (BIO6) acts as a limiting factor controlling overwinter decomposition (Matzner & Borken, 2008) [22]. Removing these variables based solely on statistical correlation would result in the loss of mechanistic drivers.

### 2.3.2 Model validation.

The dataset was partitioned into training (80%) and independent testing (20%) subsets. We implemented a hierarchical grid search to optimize hyperparameters (e.g., ntree, mtry) [14]. Model predictive accuracy was assessed on the independent test set using the Coefficient of Determination (R²) and Root Mean Square Error (RMSE) [15].

### 2.4 Spatiotemporal projection and analysis

#### 2.4.1 Space-for-time substitution.

We employed a Digital Soil Mapping (DSM) framework integrated with a space-for-time substitution paradigm. The optimized RF model, calibrated on baseline environmental relationships, was driven by the time-series climate and LULC inputs (Section 2.2.3). We generated SOC projections for four intervals: 2021–2040, 2041–2060, 2061–2080, and 2081–2100 under the selected SSP scenarios [17,18].

#### 2.4.2 Agricultural SOC dynamics analysis.

To quantify agricultural SOC dynamics, we applied a dynamic binary masking protocol. For each scenario and time-step, pixels classified as “cropland” were isolated. We calculated net SOC change by differencing future projections from the baseline. Within these constrained domains, we computed: (1) total cropland area (km²); (2) aggregate area exhibiting net SOC decline; and (3) mean SOC change.

## 3. Results

### 3.1 Descriptive statistics of baseline SOC stock

This substantial standard deviation relative to the mean highlights profound spatial heterogeneity, with maximum accumulation (8–12 kg/m²) concentrated in the southeastern physiographic region. Specifically, the humid tropical ecosystems of Chiapas, Tabasco, and eastern Veracruz exhibit the highest densities, coinciding with zones of maximum precipitation and enhanced net primary productivity.

Conversely, a systematic diminution in SOC stock characterizes the northward and westward trajectories. The central plateau functions as a transitional zone with intermediate values (4–6 kg/m²), while minimum densities (< 2 kg/m²) define the extensive arid and semi-arid northern territories, including the Baja California peninsula. This gradient demonstrates strong coherence with climatic constraints, where xeric moisture regimes in the north significantly limit organic matter accumulation compared to the moisture-rich southeast.

### 3.2 Model performance and uncertainty assessment

In this study, we extended our previous spatial analysis of soil organic carbon (SOC) stock across Mexico by employing an advanced modeling approach to elucidate the climatic drivers influencing SOC distribution. Our comprehensive analysis, based on data from 4,240 sampling points, reveals a complex interplay of key factors affecting SOC distribution, which can be broadly categorized into soil characteristics, climatic variables, and topographic factors (Fig 2). Organic Matter-Related Content (ORMC) emerged as the most significant factor, underscoring the intrinsic link between organic matter and soil carbon content and highlighting the crucial role of inherent soil properties in carbon storage processes. Bioclimatic variables followed in importance, with temperature seasonality (bio_4), annual precipitation (bio_12), and precipitation of the driest month (bio_14), and annual temperature range (bio07) identified as the most influential climatic factors. These results emphasize the critical role of climate, particularly temperature fluctuations and precipitation patterns, in shaping SOC distribution. Other bioclimatic variables, such as diurnal temperature range (bio_2) and precipitation seasonality (bio_15), while less influential, still demonstrated notable importance. Elevation, as a significant topographic factor, also exhibited substantial influence on SOC accumulation, suggesting mechanisms through which topography indirectly affects SOC distribution by modulating temperature, precipitation, and vegetation types. Cumulatively, the top 13 factors explained approximately 80% of SOC variability, providing a robust foundation for model simplification. Following our analysis of climatic drivers influencing soil organic carbon (SOC) distribution in Mexico, we employed an enhanced random forest model to simulate SOC stock under current and future climate scenarios. The model demonstrated nice predictive power (R-squared = 0.4457). Analysis of our model’s performance in predicting Soil Organic Carbon (SOC) across Mexico reveals strengths. The boxplot shows close median alignment between actual and predicted SOC values, particularly for levels below 5%, indicating accurate central tendency prediction for typical Mexican soils. This is further supported by the scatter plot, which demonstrates robust predictive capability in the 0–5% SOC range, evidenced by data points clustering near the 1:1 relationship line. However, the actual data exhibits a wider range of SOC values, including high outliers up to 30% SOC, which the model doesn’t fully capture. This suggests that while the model performs well for the arid and semi-arid regions dominating Mexico, it may underestimate SOC in carbon-rich environments like wetlands or high-altitude forests.

**Fig 3:**
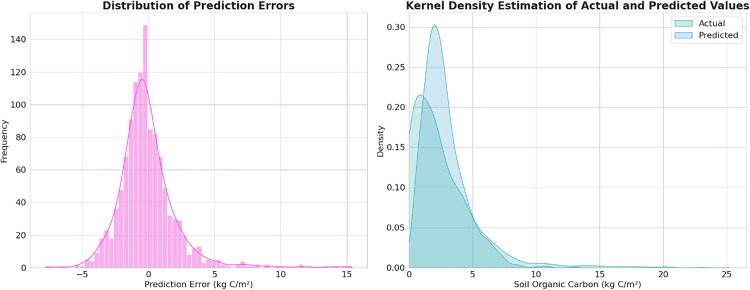
Assessment of Soil Organic Carbon (SOC) prediction model performance in Mexico about the distribution of prediction errors (A) and kernel stock estimation (B) comparing the distributions of actual and predicted SOC values across the study area.

Moreover, the error distribution and kernel stock estimation graphs provide valuable insights into our model’s performance in predicting Soil Organic Carbon (SOC) across Mexico. The distribution of prediction errors appears roughly symmetric around zero, suggesting overall unbiased predictions (Fig 3). This symmetry indicates that the model doesn’t consistently over- or under-predict SOC values, which is a positive characteristic. However, the long tails in the error distribution reveal instances of significant over- and under-predictions, particularly evident in the range of −5–5 and beyond. These outliers warrant further investigation, as they may represent specific soil conditions or ecosystems where the model’s accuracy diminishes. The kernel stock estimation graph offers a complementary perspective, showing generally good alignment between predicted and actual SOC distributions. The peak of both distributions occurs at low SOC values (around 0–5%), which aligns with the predominance of arid and semi-arid regions in Mexico. However, there’s a noticeable compression in the predicted distribution towards the mean, especially evident in the range of 5–15% SOC. This compression suggests that while the model captures the central tendency well, it may underestimate extreme values, particularly in carbon-rich environments. The model’s tendency to compress predictions towards the mean, while maintaining overall unbiasedness, indicates a trade-off between broad applicability and precision in extreme cases.

**Fig 4:**
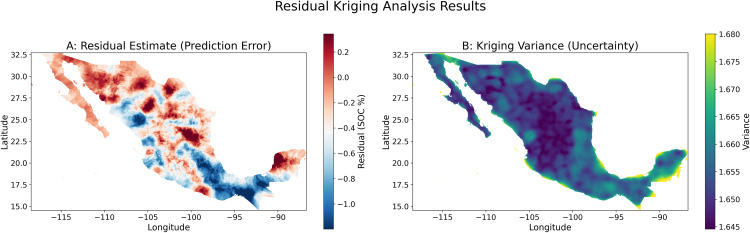
Results of the residual kriging analysis for the soil organic carbon (SOC) model residuals.

The spatial distribution of the kriged residual estimates (Fig 4A) indicates that the model’s prediction bias is not randomly distributed but exhibits distinct regional patterns. Specifically, the model tends to underestimate SOC content in extensive arid and semi-arid regions of northern Mexico (e.g., parts of Sonora and Chihuahua), certain agricultural areas in the central highlands, and northeastern portions of the Yucatán Peninsula, as evidenced by positive residuals (red-shaded areas) exceeding +0.2% SOC. Conversely, the model predominantly overestimates SOC content along the narrow western Pacific coastal strip, in the southern Sierra Madre mountain ranges, and in some humid southeastern regions, characterized by negative residuals (blue-shaded areas) approaching or even exceeding −1.0% SOC. The spatial clustering of these residuals suggests that despite the inclusion of multiple environmental covariates, uncaptured spatially dependent local environmental factors or complex interactions may still influence the actual SOC distribution, leading to systematic deviations in model predictions within specific geographic zones.

Correspondingly, the kriging variance map (Fig 4B) quantifies the uncertainty associated with these residual interpolations. This analysis shows spatial heterogeneity in the reliability of the prediction error estimates. Areas exhibiting higher kriging variance (yellow-shaded regions, variance values approaching 1.680) are predominantly located in regions where original SOC sample data are relatively sparse or potentially less spatially representative, such as remote parts of the Baja California Peninsula, some northern inland areas, and peripheral zones of the Yucatán Peninsula. In these locations, the confidence in the interpolated residual estimates is comparatively lower due to insufficient proximal observational support. In contrast, central Mexico and other well-sampled areas display lower kriging variance (dark purple regions, variance values around 1.645), indicating higher reliability of the residual estimates. The spatial pattern of kriging variance thus reflects not only the limitations of the initial sampling design but also provides crucial guidance for optimizing future sampling strategies to enhance model accuracy and reduce predictive uncertainty.

Our national-scale Soil Organic Carbon (SOC) stock prediction for Mexico (0–30 cm, Fig 1) exhibits broad spatial congruence with established global/regional products, namely the USDA-NRCS SOL_ORGANIC-CARBON_USDA-6A1C_M/v02 and ISRIC SoilGrids. All three maps consistently identify low SOC stocks in the arid northern deserts and the Baja California Peninsula. Notably, similar to these reference products, our model also predicts high SOC concentrations in the humid southeastern regions, particularly across much of the Yucatán Peninsula, Tabasco, and northern Chiapas. However, our model demonstrates enhanced spatial detail in heterogeneous landscapes and a refined delineation of SOC gradients across key ecoregions compared to the more generalized USDA product. While sharing a comparable level of spatial granularity with SoilGrids, our map presents significant regional differences in predicted SOC magnitudes; for instance, our estimates for forest areas such as the Lacandon Jungle are notably higher. This divergence is likely attributable to differences in the foundational data. It is important to acknowledge, however, that while our model captures these high SOC trends, it may, like many data-driven models, exhibit some underestimation in other specific extreme high-carbon environments such as pristine wetlands, where peak SOC values might be underrepresented in the training data or inherently challenging for the algorithm to fully capture. Despite this caveat, these comparative insights underscore the value of region-specific modeling in refining SOC inventories and understanding local drivers.

### 3.3 Spatiotemporal projections of SOC stock under future climate SSPs

The temporal evolution of mean SOC stocks reveals a critical divergence between sustainable development pathways and fossil-fueled scenarios (Table 1). Under the SSP126, SSP245, and SSP370 scenarios, national mean SOC stocks exhibit a resilient trajectory. These pathways project an initial increase to approximately 3.06 kg C/m^2^ during the 2021–2040 period, followed by a stabilization around 3.02 kg C/m^2^ through 2100. Crucially, these scenarios consistently maintain SOC levels approximately 2% above the current baseline (2.96 kg C/m^2^) throughout the 21st century. In stark contrast, the SSP585 scenario projects a concerning reversal. Despite a transient increase in the near term, mean SOC stocks under this high-emission pathway enter a continuous decline after 2040, falling to 2.94 kg C/m^2^ by the 2061–2080 period. This trajectory represents a potential functional shift, where the initial sequestration gains are negated by intense climatic forcing, driving the national mean below the current baseline by the latter half of the century.

**Table 1:** Soil organic carbon stock predictions for mexico under different climate scenarios (Unit: kg/SOC/m^2^).

Period	Current	2021-2040	2041-2060	2061-2080	2081-2100
Current	2.96 ± 1.82	—	—	—	—
SSP126	—	3.06 ± 1.86*	3.03 ± 1.84	3.02 ± 1.82	3.02 ± 1.82ᵃ
SSP245	—	3.06 ± 1.86*	3.03 ± 1.84	3.02 ± 1.82	3.02 ± 1.82ᵃ
SSP370	—	3.06 ± 1.86*	3.03 ± 1.84	3.02 ± 1.82	3.02 ± 1.82ᵃ
SSP585	—	3.03 ± 1.84*	3.00 ± 1.83	2.94 ± 1.79	2.96 ± 1.82ᵃ

**Fig 5:**
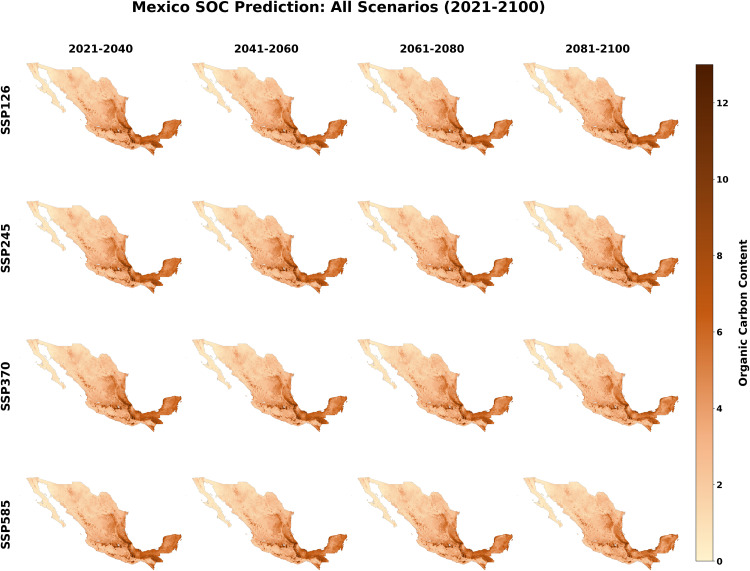
Spatiotemporal projections of Soil Organic Carbon (SOC) stocks in Mexico under different Shared Socioeconomic Pathways (SSPs) from 2021 to 2100.

Spatially, the projected SOC distribution across all four scenarios maintains the foundational gradient governed by climatic and physiographic constraints (Figs 5–). The highest concentrations (8 ~ kg C/m^2^) persist in the humid southeastern states (e.g., Chiapas, Tabasco) and the Gulf Coast, while the lowest stocks (<2 ~ kg C/m^2^) characterize the arid northern deserts and the Baja California Peninsula. However, the intensity and extent of these patterns vary significantly by pathway. Under the sustainable SSP126 scenario, the high-SOC zones in the southeast and east-central regions exhibit resilience and a slight expansion, particularly in transition zones, reflecting conditions conducive to continued sequestration. Conversely, under SSP585, these carbon-rich “hotspots” show signs of contraction and intensity loss in the late century (2081–2100). The stabilization observed in SSP245 and SSP370 represents an intermediate state, further confirming that future SOC stability is strictly pathway-dependent, with the most severe degradation risks concentrated in currently carbon-rich ecosystems under high-emission futures.

**Fig 6:**
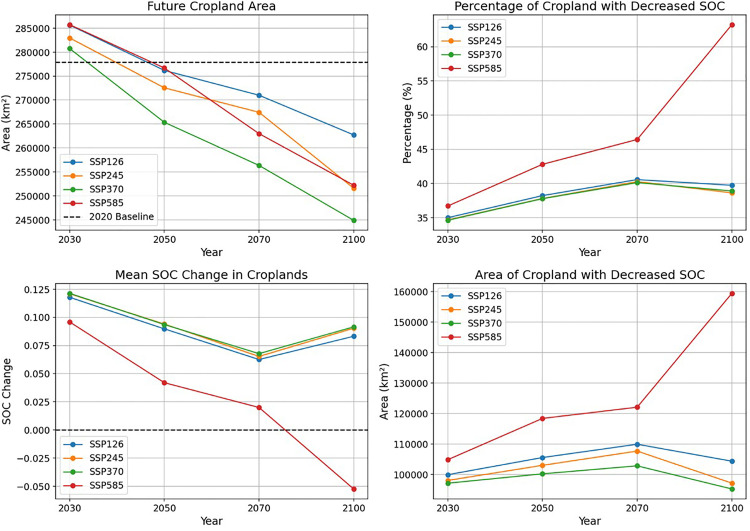
Divergent trajectories of cropland area and soil organic carbon (SOC) in Mexico under Shared Socioeconomic Pathways (SSPs).

**Fig 7:**
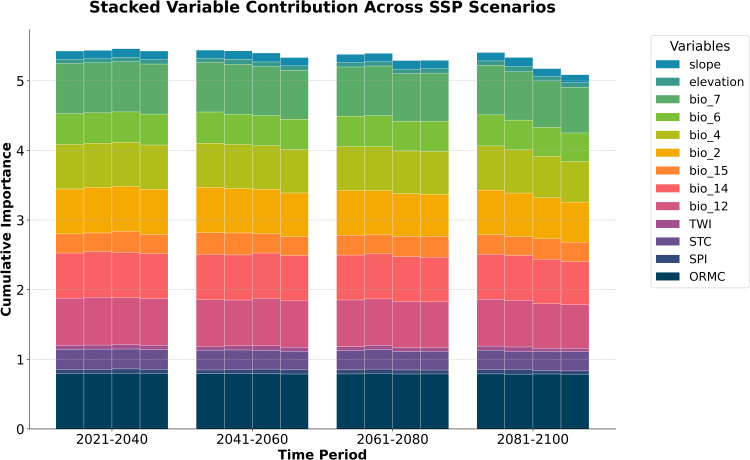
The importance predictor analysis of soil organic carbon (SOC) dynamics. Detailed descriptions of the variables are provided in the notes below the figure.

### 3.4 Contraction of cropland area and divergent degradation trajectories

According to the study of land use prediction data under different SSP scenarios by Zhang et al. (2023) pathway-dependent contraction of Mexico’s total cropland area from 2030 to 2100. [10] Following a brief expansion, the cropland area in all scenarios falls below the 2020 baseline (277,905 km²) by mid-century (Fig 6A). The most severe attrition is projected under SSP370 (Regional Rivalry), with an 11.9% reduction by 2100, while SSP126 (Sustainability) exhibits the greatest resilience with only a 5.5% loss. Concurrently, the proportion of cropland experiencing a net decline in soil organic carbon reveals two distinct patterns. For scenarios SSP126, SSP245, and SSP370, this proportion stabilizes around 40% after peaking mid-century. In stark contrast, the SSP585 (Fossil-fueled Development) scenario exhibits a continuous and accelerating increase, with the fraction of degrading cropland rising from 36.7% in 2030 to 63.2% by 2100 (Fig 6B). As shown in Fig 6C, the mean soil organic carbon change remains positive throughout the 21st century for the SSP126, SSP245, and SSP370 scenarios, establishing the system as a net carbon sink. In stark contrast, the SSP585 scenario projects a precipitous decline, with the system crossing a zero-threshold post-2070 to become a net carbon source. This functional shift is corroborated by Fig 6D, which shows a near-exponential expansion of degraded land area under SSP585, reaching 159,464 km² by 2100—a scale of increase significantly larger than in any other scenario.

## Discussion

### 4.1 Dominant drivers of future soil organic carbon dynamics

Analysis of predictor importance (Fig 7) reveals a consistent, hierarchical control over projected soil organic carbon dynamics. Land Use/Land Cover (LULC) and initial SOC stock (ORMC) emerge as dominant foundational drivers, their substantial and persistent contributions throughout the 21st century underscoring the critical modulation of future SOC trajectories by anthropogenic landscape alteration and inherent soil carbon path dependency. This national-scale finding aligns strongly with regional field studies in Mexico, which identify the legacy effects of land-use history and the inherent time lags of ecological succession as key determinants of carbon dynamics [11,12]. Collectively, climatic variables exert the next tier of significant influence, with precipitation-related bioclimatic indicators (bio_12, bio_14, bio_15) demonstrating greater cumulative importance than temperature-related ones (bio_2, bio_4, bio_7), highlighting regional moisture regimes as paramount climatic regulators of SOC. The mechanism underlying this finding is elucidated by the work of Cuevas et al. (2013) [13], who confirmed that in seasonally dry ecosystems, precipitation primarily modulates the net SOC balance by regulating microbial decomposition rates, rather than through a simple, positive linear relationship. While elevation contributes consistently, its impact is secondary. Subtle temporal shifts in the relative importance of specific bioclimatic variables, alongside a slight decrease in overall cumulative variable importance by 2081–2100, may suggest evolving SOC sensitivities to different facets of climate change and potentially increased predictive uncertainty in the far future.

It is important to acknowledge the limitations of the Random Forest model, particularly its performance in high-carbon environments. As an interpolation-based algorithm, RF is constrained by the range of the training data, which can create a “predictive ceiling” and lead to the underestimation of potential SOC gains in high-stock areas. To address this, future research should explore alternative or hybrid modeling strategies. For instance, Quantile Regression Forests could better capture prediction uncertainty and extreme values, while coupling our data-driven approach with process-based models (e.g., CENTURY, RothC) would enhance mechanistic realism and improve forecasting in these critical ecosystems.

### 4.2 Divergent trajectories of cropland SOC dynamics across SSP scenarios and underlying drivers

This study reveals markedly divergent trajectories for future S dynamics in Mexican croplands under different Shared Socioeconomic Pathways (SSPs) (Fig 8). Notably, under SSP126 (Sustainability), SSP245 (Middle of the Road), and SSP370 (Regional Rivalry), despite a projected contraction in total cropland area (Fig 8A), the mean SOC change across Mexican croplands is projected to remain positive throughout the 21st century, indicating that these systems could function as net carbon sinks (Fig 8C). Within these scenarios, although approximately 40% of croplands experience SOC decline post mid-century (Fig 8B), the extent of SOC degradation does not exhibit continued expansion, suggesting a degree of system resilience. This relatively positive outlook likely stems from a complex interplay of factors. For instance, the SSP126 scenario, with its strong emphasis on global sustainability goals, may translate into proactive national policies promoting conservation agriculture, organic inputs, and soil erosion control measures in Mexico, thereby enhancing soil carbon inputs and minimizing losses. Crucially, the contraction of cropland under this scenario likely allows marginal lands to enter natural recovery pathways, where soil C and N dynamics can be restored over decades, as documented by the  60-year recovery timeline reported by Saynes et al. (2005). [14] Even under SSP370, which presents greater socioeconomic challenges and projects the most significant cropland area loss (Fig 8A), the persistence of a net positive mean SOC change is intriguing. This could potentially be attributed to the abandonment of marginal, low-SOC croplands, with retained core agricultural areas experiencing SOC gains under specific (possibly intensified or improved) management practices. However, the “Regional Rivalry” narrative of SSP370 also warrants caution regarding the long-term sustainability of soil health if short-term, high-input agricultural intensification prevails. [15] The stark contrast between these scenarios and SSP585 underscores the critical modulating influence of socioeconomic development pathways on regional carbon cycling. In sharp contrast to the aforementioned scenarios, SSP585 (Fossil-fueled Development) portends a precipitous deterioration of cropland SOC in Mexico. Under this pathway, not only does the proportion of cropland experiencing SOC decline escalate from 36.7% in 2030 to 63.2% by 2100 (Fig 8B), but more critically, the mean SOC change for Mexican croplands transitions from positive to negative post-2070, shifting the entire system from a net carbon sink to a significant net carbon source (Fig 8C). This functional shift is accompanied by a near-exponential expansion of degraded land area, reaching 159,464 km² by 2100 (Fig 8D), posing a severe threat to regional carbon balance, ecosystem services, and agricultural sustainability. The unique severity of SSP585 arises from its coupling of the most intense climate change impacts, driven by high greenhouse gas emissions, [16] with a socioeconomic development model heavily reliant on fossil fuels and potentially neglecting environmental safeguards. The projected dramatic decline in cropland SOC under these conditions can be attributed to: (1) significantly accelerated decomposition rates of soil organic matter due to extreme warming and potential aridification trends; [17,18] (2) unsustainable agricultural practices, such as intensive tillage, inadequate organic matter return, and mismanagement of water resources, further exacerbating SOC losses; [19] and (3) a vicious cycle of land degradation, where SOC depletion impairs soil structure and water retention, thereby reducing plant productivity, diminishing organic inputs, and further accelerating SOC exhaustion. [20] The projections under SSP585 serve as a stark warning for Mexico and other ecologically vulnerable regions globally: without aggressive climate mitigation and transformative socioeconomic shifts, high-emission, unsustainable development pathways are likely to reverse the carbon sink capacity of terrestrial ecosystems, critically undermining carbon neutrality goals and severely jeopardizing regional food security and environmental health.

## Conclusion

This study elucidates the spatiotemporal dynamics of soil organic carbon in Mexico, identifying a profound spatial heterogeneity driven by climatic gradients and land use. Our projections confirm that while southeastern humid regions act as critical carbon reservoirs, the arid northern territories remain naturally carbon-depleted.

The most significant finding is the stark divergence in future SOC trajectories determined by socioeconomic choices. Under sustainable and intermediate pathways (SSP126, SSP245, SSP370), national SOC stocks demonstrate resilience, stabilizing or slightly increasing (~2% above baseline) by 2100. In contrast, the fossil-fueled development pathway (SSP585) triggers a functional regression, driving mean SOC stocks below current baseline levels and causing substantial losses in carbon-rich hotspots.

Crucially, this divergence is most acute in agricultural systems. Even under sustainable scenarios, approximately 40% of cropland is projected to face degradation; however, the sector as a whole maintains resilience, functioning as a net carbon sink. In dramatic contrast, the SSP585 pathway precipitates a catastrophic shift. Under this high-emission scenario, the proportion of degrading cropland escalates to 63.2%, transitioning Mexico’s agricultural system from a net sink to a net carbon source after 2070. This impending “sink-to-source” transition underscores that future soil stability is strictly policy-dependent, necessitating immediate intervention to safeguard national carbon neutrality goals.

### Notes

slope: Surface inclination, representing the steepness of the terrain.

elevation: Vertical height above sea level.

bio_2 (Mean Diurnal Range): Average difference between daily maximum and minimum temperatures.

bio_4 (Temperature Seasonality): Standard deviation of monthly temperature, indicating temperature variability.

bio_6 (Min Temperature of Coldest Month): Mean minimum temperature in the coldest month.

bio_7 (Temperature Annual Range): Difference between maximum temperature of the warmest month and minimum temperature of the coldest month.

bio_12 (Annual Precipitation): Total annual precipitation.

bio_14 (Precipitation of Driest Month): Mean precipitation in the driest month.

bio_15 (Precipitation Seasonality): Coefficient of variation of monthly precipitation, indicating precipitation variability.

TWI (Topographic Wetness Index): An index of terrain’s propensity to accumulate water, reflecting potential soil moisture.

STC (Soil Texture Class): Describes the relative proportions of sand, silt, and clay particles in soil.

SPI (Stream Power Index): An index representing the erosive power of flowing water.

ORMC (Organic Matter Content): The percentage of decomposed organic material in the soil.
